# Effect of using electronic medication monitors on tuberculosis treatment outcomes in China: a longitudinal ecological study

**DOI:** 10.1186/s40249-021-00818-3

**Published:** 2021-03-17

**Authors:** Ni Wang, Lei Guo, Hemant Deepak Shewade, Pruthu Thekkur, Hui Zhang, Yan-Li Yuan, Xiao-Meng Wang, Xiao-Lin Wang, Miao-Miao Sun, Fei Huang, Yan-Lin Zhao

**Affiliations:** 1 National Center for Tuberculosis Control and Prevention, Chinese Center for Disease Control and Prevention, Beijing, China; 2 Duke Global Health Institute, Duke University, Durham, NC USA; 3 International Union Against Tuberculosis and Lung Disease (The Union), Paris, France; 4 The Union South-East Asia Office, New Delhi, India; 5 Jilin Research Institute of Tuberculosis Control, Changchun, China; 6 Zhejiang Province Center Disease Control and Prevention, Hangzhou, China; 7 The Fourth People’s Hospital of Ningxia Hui Autonomous Region, Yinchuan, China; 8 Program for Appropriate Technology in Health(PATH), China Program, Shanghai, China

**Keywords:** Tuberculosis, Treatment outcome, Medication monitoring, Digital technology, Longitudinal study

## Abstract

**Background:**

In China, an indigenously developed electronic medication monitor (EMM) was designed and used in 138 counties from three provinces. Previous studies showed positive results on accuracy, effectiveness, acceptability, and feasibility, but also found some ineffective implementations. In this paper, we assessed the effect of implementation of EMMs on treatment outcomes.

**Methods:**

The longitudinal ecological method was used at the county level with aggregate secondary programmatic data. All the notified TB cases in 138 counties were involved in this study from April 2017 to June 2019, and rifampicin-resistant cases were excluded. We fitted a multilevel model to assess the relative change in the quarterly treatment success rate with increasing quarterly EMM coverage rate, in which a mixed effects maximum likelihood regression using random intercept model was applied, by adjusting for seasonal trends, population size, sociodemographic and clinical characteristics, and clustering within counties.

**Results:**

Among all 69 678 notified TB cases, the treatment success rate was slightly increased from 93.5% [95% confidence interval (*CI*): 93.0–94.0] in second quarter of 2018 to 94.9% (95% *CI*: 94.4–95.4) in second quarter of 2019 after implementing EMMs. There was a statistically significant effect between quarterly EMM coverage and treatment success rate after adjusting for potential confounders (*P* = 0.0036), increasing 10% of EMM coverage rate will lead to 0.2% treatment success rate augment. Besides, an increase of 10% of elderly or bacteriologically confirmed TB will lead to a decrease of 0.4% and 0.9% of the treatment success rate.

**Conclusions:**

Under programmatic settings, we found a statistically significant effect between increasing coverage of EMM and treatment success rate at the county level. More prospective studies are needed to confirm the effect of using EMM on TB treatment outcomes. We suggest performing operational research on EMMs that provides real-time data under programmatic conditions in the future.

**Figure:**
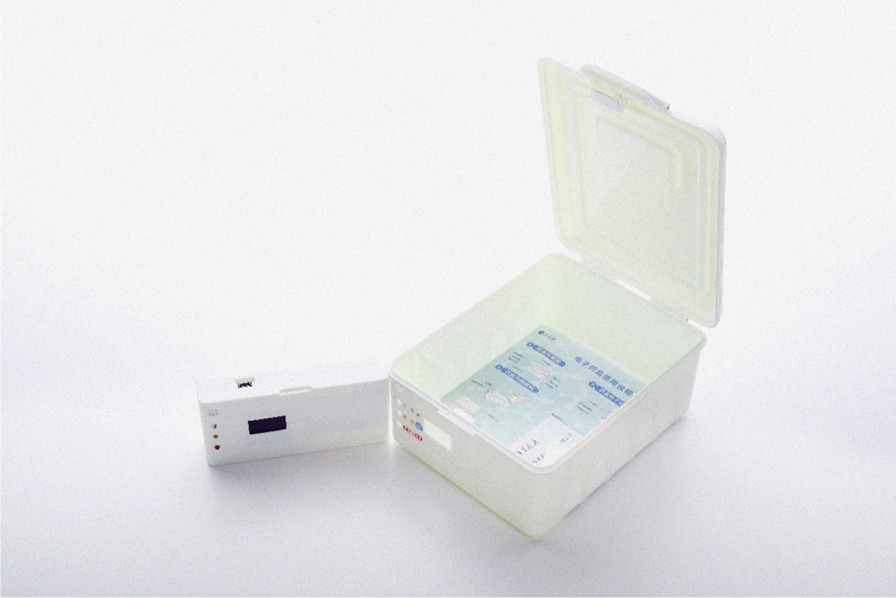

**Supplementary Information:**

The online version contains supplementary material available at 10.1186/s40249-021-00818-3.

## Background

Globally, an estimated 9.96 million people fell ill with tuberculosis (TB) in 2019, a number that has been relatively stable in recent years [1]. China has the world’s third highest TB burden, with an estimated 833 000 patients in 2019, accounting for 8.4% of the global burden [1]. The treatment success rate for new and relapse cases was 85% (2018 cohort) worldwide [1]. Nonadherence was the most significant risk factor for an unfavourable outcome, and missing 10% of doses or more was associated with an adjusted hazard ratio of 5.7 (95% confidence interval [*CI*]: 3.3–9.9) [2].

The World Health Organization (WHO) recommends patient-centred care with feasible treatment administration options [3]. Although the model of directly observed therapy (DOT) by health care workers has been optimized for community-based or home-based DOT, and the DOT provider could be a health care worker, lay provider, or family member, it is still impossible to universally implement DOT in a country like China with its heavy TB burden. A systematic review found that about 52% of TB patients adopted self-administered therapy (SAT) in China[4]. Unfortunately, when compared with DOT alone, SAT was associated with lower rates of treatment success, adherence, and sputum smear conversion as well as higher rates of development of drug resistance [5, 6]. To address these challenges, digital health technologies like short message service (SMS, or text messaging), electronic medication monitors (EMMs), and video-observed treatment are the potential adherence support mechanisms [7]. A study from India estimates that using these new technologies could have a substantial impact on TB, decreasing the incidence by 16% between 2020 and 2030, if deployed optimally in both public and private sectors [8].

EMMs aim to provide more patient flexibility by supporting patients with instructions, dosing alerts, and refill reminders. These devices enable recording patient-specific dosing histories, as this provides the most accurate and detailed insights into patients’ behavior in taking medication, although EMMs are still an indirect method of measuring treatment adherence [9]. A systematic review found that EMMs were associated with lower rates of loss to follow-up [relative risk (*RR*) = 0.59, 95% *CI*: 0.43–0.80], poor outcome (*RR* = 0.63, 95% *CI*: 0.47–0.83), and poor adherence (*RR* = 0.57, 95% *CI*: 0.53–0.61) [5]. Compared with other digital technologies, EMMs are relatively simple in technology and inexpensive.

In China, an indigenously developed EMM (that does not provide real-time data) was designed and scaled up gradually. Under trial conditions, the EMM decreased the frequency of missed doses by 40% to 50% compared to SAT [10]. Under programme settings, the EMM showed a high level of acceptability and satisfaction among the people with TB and health care workers, while children (< 15 years), elderly people(≥ 65 years), semi-skilled or unemployed people, people with tuberculosis pleurisy and previous tuberculosis treatment were less likely to use EMM [11, 12]. Besides, instances of missing EMM data in the information management system (EMMIMS) were common (25.1%). Furthermore, in nearly four-fifths of the instances, people were not shifted to DOT although objective evidence of nonadherence was available [13]. Under an intention-to-treat analysis from 30 counties in China, EMM did not result in improved TB treatment outcomes when compared to SAT alone [14]. However, there are still limited studies assessing the effect of implementation of EMMs on treatment outcomes [15, 16].

In this paper, we aimed to study whether introducing EMM under programme settings and increasing its coverage results in improved TB treatment outcomes at the county level in China.

## Methods

### Study setting

#### TB treatment and management

In China, diagnosed TB patients are routinely notified in a web-based TB information management system (TBIMS). Patients not known to be rifampicin resistant will receive daily fixed-dose treatment over six to eight months depending on whether the patient is new or previously treated (see Additional file 1 for details of the treatment regimens). According to China’s national TB programme (NTP) for treatment adherence management, TB patients could receive DOT from village doctors (village-level licensed general practitioners), family members, or volunteers, if conditional. Otherwise, they will receive SAT with or without support by digital adherence technologies. The village doctors are expected to visit patients every 10 days during the first two months of treatment followed by once a month to assess their adherence. Irrespective of DOT or SAT, patients have to visit the TB designated hospitals at the county level (TB basic management unit) once every month for a checkup on their health status and to refill the medicine.

#### EMM scale up plan and study sites

The EMM was designed to provide a medication reminder throughout a 1-month fixed-dose regimen, and it records each time the patient opens the device, indicating the patient has taken his or her medication. The structure, function, and quality control protocol of the EMM have been described in the previous study [13] (see Additional file 2 for the picture of the EMM device). Under the support of the National Health Commission of the People's Republic of China–Bill & Melinda Gates Foundation Tuberculosis Prevention and Control Project (China-Gates Foundation TB Project), the Chinese Center for Disease Control and Prevention (China CDC) developed an EMM scale-up plan for three provinces involving 138 counties: 65 from Zhejiang Province (eastern region), 51 from Jilin Province (middle region), and 22 from Ningxia Autonomous Region (west region) (see Additional file 3 for details). Implementation started in June 2018 in a phase-wise manner, and by January 2019, all the 138 counties started using EMMs (see Additional file 4 for details).

According to the geographical location and economic development level, China is divided into eastern, middle and west regions. The level of economic development decreases sequentially from east to west, the per capita gross domestic product in 2018 was United State Dollar (USD) 15 577, USD 8508 and USD 8276 in Zhejiang, Jilin and Ningxia from the website of the provincial bureau of statistics. Meanwhile, the prevalence of TB is higher in the western region compared to the eastern and middle regions[17]. Given the significant differences among those counties, the cluster at the county level was considered to manage the data set.

#### EMM to support TB management

During each time the patients visited TB designated hospitals at the county level, doctors generated the adherence report by connecting the EMM to an offline software program in their computers. If < 20% of doses were missed, the person was counselled on the importance of treatment adherence. If 20%–49% of doses were missed, the frequency of home visits by village doctors was increased to once every seven days for the rest of the treatment. If there was continued instance of missing 20%–49% of doses or a single instance of missing ≥ 50% of doses, people were shifted to DOT administered by village doctors. Without using EMM, doctors would assess a patient’s adherence based on his or her treatment card by patient self-report, and the same actions would be taken as described previously, although it was widely believed to overstate actual adherence [18].

### Study design and population

This was a longitudinal ecological study (stepped-wedge design) involving county-level aggregate secondary programmatic data [19].

The study population included the notified TB patients in the 138 counties from three provinces in China, between April 2017 and June 2019. TB patients (not known to be rifampicin resistant) having no communication impairment (mental, visual, auditory, or speech) would be suitable to use EMM. All the eligible TB patients were suggested to use EMM at the beginning of treatment. The verbal consent of using EMM was obtained by physicians at the TB designated hospitals.

There were eight TB designated hospitals responsible for more than two counties. We couldn’t distinguish the number of patients from them, so we combined these counties that shared the same TB designated hospital as a single unit.

The first four quarters (second quarter of 2017–first quarter of 2018) will represent the pre-implementation period when all 138 counties were not implementing EMMs. The last five quarters (second quarter of 2018–second quarter of 2019) will represent the implementation period. Within the implementation period, the first three quarters (second quarter–fourth quarter of 2018) were when the phase-wise implementation happened, and in the last two quarters (first quarter–second quarter of 2019), all the counties were implementing EMMs.

### Data sources and variable definitions

Quarterly aggregate county-level data were collected over nine quarters (second quarter–fourth quarter of 2017, first quarter–fourth quarter of 2018, and first quarter–second quarter of 2019). We extracted data variables from TBIMS (county ID, sex, age, occupation, migrant status, category of TB, classification of TB, drug susceptibility test results, treatment outcomes), EMMIMS (whether use of EMM, number of TB patients started on EMM), and paper-based investment (number of TB designated hospitals in the county, when was GeneXpert facility available in the county). County populations were extracted from the Bureau of Statistics website of each province.

We classified cure and treatment completion as favourable treatment outcomes or treatment success, and loss to follow up, death, treatment failure, and not evaluated as unfavourable outcomes (see Additional file 5 for details of the definition). All the treatment outcomes data were collected at least one year after the notification.

### Statistical analysis

We used SAS software (version 9.4, SAS Institute Inc. Cary, NC) for analysis. Among notified TB patients, we calculated the following proportions quarterly, county wise: number of TB patients started on EMMs, phenotypic drug susceptibility tests done, molecular drug susceptibility tests done, males, elderly (≥ 65 years), unemployed, migrant, previously treated, bacteriologically confirmed, pulmonary TB, and TB treatment success.

We described the treatment success rates quarterly (second quarter of 2017–second quarter of 2019) by counties, stratified by EMM using status and the percentage of EMM used in the quarter. Cochran-Armitage test was used to test the trend difference. We also fitted a multilevel model (mixed effects maximum likelihood regression using random intercept model) to assess the relative change in the quarterly treatment success proportion with increasing quarterly EMM coverage rates, considering the clustering at the county level, by adjusting the seasonal trends, the population size of the counties, sociodemographic and clinical characteristics, and other potential confounders at the county level. We used the Durbin-Wu-Hausman endogeneity test to assess whether the random intercept model must be retained (if the Durbin-Wu-Hausman test is nonsignificant (*P* > 0.05)).

## Results

### Patient characteristics

A total of 69 678 people were notified with TB between April 2017 and June 2019, and among them, 17 327 (24.9%) were elderly, 47 650 (68.4%) were male, 12 858 (18.5%) were unemployed, and 12 584 (18.1%) were staying in the same prefecture for less than six months. In addition, 59 770 (85.8%) were pulmonary TB patients, 32 540 (46.7%) were bacteriologically confirmed TB, and 5 178 (7.4%) were previously treated for TB. (Table1).

**Table 1:** Sociodemographic and clinical characteristics of notified cases between April 2017 and June 2019 in 138 counties

Characteristics	*N*	(%)
Total	69 678	100.0
Sociodemographic		
Elderly (≥ 65 years)		
No	52 351	75.1
Yes	17 327	24.9
Gender		
Male	47 650	68.4
Female	22 028	31.6
Occupation		
Unemployed	12 858	18.5
Employed	56 820	81.5
Migrant^a^		
Yes	12 584	18.1
No	57 094	81.9
Clinical		
PTB		
Yes	59 770	85.8
No	9908	14.2
Classification		
Bacteriologically confirmed	32 540	46.7
Clinically diagnosed	37 138	53.3
Category		
New	64 500	92.6
Previously treated	5178	7.4
Testing for DR-TB		
Not testing	41 009	58.9
PDST	8949	12.8
MDST	19 720	28.3

Column percentages

*EMM* Electronic medication monitor, *DR-TB* Drug resistance TB, *PDST* Phenotypic drug susceptibility tests, *PTB* Pulmonary TB, *MDST* Molecular drug susceptibility tests, *TB* Tuberculosis

^a^Migrant defined as person staying in the same prefecture for less than 6 months

### Treatment outcomes stratified by EMM status and coverage

The steps of EMM implementation are depicted in Table 2. EMM implementation started in June 2018 and was scaled up using a stepwise approach in the three provinces. Finally, all the 138 counties started to implement EMM in January 2019.

**Table 2:** Favourable treatment outcomes stratified by EMM status at the county level quarterly in 138 counties

Time	EMM counties^a^		Non-EMM counties	
	Number of counties	Favourable treatment outcome^b^ *N* (%)	Number of counties	Favourable treatment outcome^b^ *N* (%)
2017 Q2	0	-	138	8255/8731 (94.5)
2017 Q3	0	-	138	7599/8041 (94.5)
2017 Q4	0	-	138	6873/7289 (94.3)
2018 Q1	0	-	138	6616/7036 (94.0)
2018 Q2	33	1451/1565 (92.7)	105	6854/7320 (93.6)
2018 Q3	89	4612/4934 (93.3)	49	2788/2938 (94.9)
2018 Q4	129	6064/6427 (94.4)	9	467/496 (94.2)
2019 Q1	138	6702/7104 (94.3)	0	-
2019 Q2	138	7388/7788 (94.9)	0	-

Line percentages

*EMM* Electronic medication monitor, *Q* Quarter

^a^At least one patient used EMM in the county within one quarter

^b^Favourable treatment outcomes defined as the sum of cured and treatment completed

EMM implementation began in June 2018, and then was scaled up using a stepwise approach in the three provinces. Finally, all the 138 counties started to implement EMM in Jan 2019

**Fig. 1:**
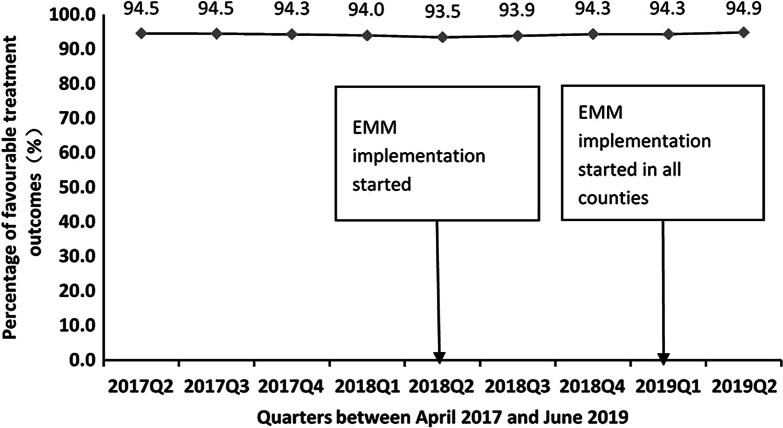
Trend for percentage of favourable treatment outcomes^*^ of notified cases between April 2017 and June 2019 in 138 counties. EMM: Electronic medication monitor. * Favourable treatment outcomes defined as the sum of cured and treatment completed. EMM implementation began June 2018, and was scaled up using a stepwise approach in the three provinces. Finally, all the 138 counties started to implement EMM in Jan 2019

Before implementing EMM, the percentage of favourable treatment outcomes was above 94% (95% *CI*: 94.1–95.0) from the 138 counties. The favourable treatment outcomes rate slightly increased from 93.5% (95% *CI*: 93.0–94.0) to 94.9% (95% *CI*: 94.4–95.4) after introducing EMMs, but there was not a statistically significant trend of change (*P* = 0.809). (Fig. 1.)

The treatment outcomes, stratified by the percentage of EMMs used quarterly for each county, are depicted in Table 3. Among the counties that used EMM in less than 30% of notified TB cases in the quarter, favourable treatment outcome was 94.0% (95% *CI*: 93.5–94.6), compared to 94.5% (95% *CI*: 94.1–94.9) in the counties that used EMM above 60% in the quarter, but there is a lack of significant difference among these grades (*P* = 0.873).

**Table 3:** Treatment outcomes stratified by the percentage of EMM use at the county level quarterly in 138 counties

Percentage of EMM use at county level quarterly (%)	Number of patients	Treatment outcomes			
		Favourable^a^		Unfavourable^b^	
		*n*	(%)	*n*	(%)
0	42 550	40 117	94.3	2433	5.7
1–29	7184	6756	94.0	428	6.0
30–59	7075	6640	93.9	435	6.1
≥ 60	12 869	12 156	94.5	713	5.5
Total	69 678	65 669	94.2	4009	5.8

Line percentages

*EMM* electronic medication monitor

^a ^Favourable treatment outcomes defined as the sum of cured and treatment completed

^b ^Unfavourable treatment outcomes defined as all outcomes other than cured and treatment completed

### Multilevel model for assessing the treatment outcomes

Durbin-Wu-Hausman endogeneity test proved the random intercept model should be retained (*P* > 0.05). There was a statistically significant effect between increasing coverage of EMMs and treatment success proportion at the county level, after adjusting for potential confounders (*P* = 0.0036). An increase of 10% EMM coverage rate will lead to an augment of 0.2% of treatment success rate. (Table 4).

**Table 4:** Multilevel model to assess the association between the percentage of EMM use and treatment success rate

Parameter	Coefficient	Standard error	*t* value	*P* value
Intercept	88.5483	3.9113	22.64	< 0.0001*
Percent EMM	0.0206	0.0071	2.92	0.0036*
(Quarter-1)	0.4369	0.5536	0.79	0.4302
(Quarter-2)	0.6990	0.4944	1.41	0.1577
(Quarter-3)	0.6088	0.5173	1.18	0.2395
(Quarter-4)	Ref	-	-	-
(TB desighosp-1)	3.5076	3.3611	1.04	0.2969
(TB desighosp-2)	2.3195	3.5535	0.65	0.5141
(TB desighosp-4)	Ref	-	-	-
County population	0.0317	0.0108	2.95	0.0033*
Xpert	– 0.4626	0.4072	– 1.14	0.2563
Percent pdst	0.0418	0.0250	1.67	0.0949
Percent mdst	0.0366	0.0229	1.60	0.1105
Percent elderly	– 0.0417	0.0173	– 2.42	0.0159*
Percent male	0.0274	0.0161	1.71	0.0883
Percent unemployed	0.0118	0.0124	0.95	0.3436
Percent prevtreat	0.0027	0.0320	0.08	0.9340
Percent bact confirm	– 0.0913	0.0250	– 3.65	0.0003*
Percent ptb	0.0188	0.0150	1.25	0.2114
Percent migrant	– 0.0054	0.0138	– 0.39	0.6962

Variability	Variance component	Standard error	*Z* value	*P* value
ζ(county)^a^	2.1801	0.4993	4.37	< 0.0001*
Residual	15.1273	0.6680	22.65	< 0.0001*

*EMM* Electronic medication monitor, *TB* Tuberculosis

Parameter abbreviations: Percent EMM, percentage of EMM used; TB desighosp, the number of TB designated hospital in the county; County population, annual population of the county; Xpert, whether GeneXpert facility in the county; Percent pdst, percentage of phenotypic drug susceptibility tests done; Percent mdst, percentage of molecular drug susceptibility tests done; Percent elderly, percentage of elderly TB cases; Percent male, percentage of male TB cases; Percent unemployed, percentage of unemployed TB cases; Percent prevtreat, percentage of previously treated TB cases; Percent bact confirm, percentage of bacteriologically confirmed TB cases; Percent ptb, percentage of pulmonary TB cases; Percent migrant, percentage of migrant TB cases

^*^P < 0.05

^a ^The random intercept for county level

Among the potential confounders, we found statistically significant association on the percentage of elderly (*P* = 0.0159) and percentage of bacteriologically confirmed TB within notified TB persons (*P* = 0.0003). An increase of 10% of elderly or bacteriologically confirmed TB will lead to a decrease of 0.4% and 0.9% of the treatment success rate. (Table 4).

## Discussion

This is a longitudinal ecological study to investigate the effect of EMM that didn’t provide real-time data under programmatic conditions. In addition, the study was conducted in representative provinces from the east, middle, and west of China, and could be used to inform the development of health policy regarding to TB care and management.

### Key findings

Our findings suggest that TB treatment outcome was improved, with the increased coverage of EMM among TB patients at the county level under programmatic conditions, and that the treatment success rate would increase by 1.5% when EMM coverage reached 75% after adjusting potential confounders. In China, the latest survey indicated that about 73% of the eligible people had ever used EMM according to patients’ consent under programmatic condition [13].

### Interpretation of key findings

Our findings are consistent with other EMM studies that provided real-time data. In rural Morocco, EMM (that provide a real-time data) increased treatment success and decreased the lost to follow-up among people with new smear positive pulmonary TB when compared to SAT alone [20]. Another study in South Africa, using SIMpill system (real-time data communicated with a web-based application by SMS every time the patient opens the bottle), also found that TB cure rates improved compared with the control group. No matter what kind of EMMs, the medication reminder for people with TB is similar; the difference lies in the timeliness of interventions from health providers. We believe the results observed in this study are plausible, taking into account the positive results from previous studies on accuracy [21], effectiveness [10], acceptability [11, 13], and feasibility [12].

Meanwhile, our findings are in contrast to our previous study, which involved 30 counties and used intention-to-treat analysis. The sample size was powered enough only to detect a minimum 50% relative reduction in unfavourable outcomes, and intention-to-treat analysis gives conservative estimates also [14]. In this study, 138 counties were involved with a large number of samples, we have 99% power to detect a minimum of 0.4% relative increase in favourable outcomes. But evidence of the effect of digital technologies to improve TB care remains limited, especially on the EMMs that do not provide real-time data. A published protocol demonstrated that a similar EMM is under implementation in a randomized clinical trial in China, but the results have not been published [22].

Although we found the EMM resulted in improved TB treatment outcomes when compared to SAT alone, the treatment success rate increased slightly. We have described in the previous study that introducing EMM was just one of the many interventions of comprehensive TB control model, designed by the China-Gates Foundation TB Project, and ineffective implementation could reduce the effect of the intervention also [14]. A study from south India showed TB treatment outcomes even conversely worsened after using 99DOTS platform (using drug packaging to facilitate contact between a TB patient and a call centre for ongoing adherence support) due to ineffective implementation [23]. In addition, study under a programme setting may weaken the effect of the intervention, but the results are more realistic. Besides, the treatment success rate is pretty high in new and relapse cases from China, reaching 94% in a 2018 cohort nationwide [1].

Of course, we should notice that implementation of EMM could also significantly reduce the workload of DOT providers by reducing patient visits by 87.9% [11]. Moreover, COVID-19 has brought substantial challenges to TB control. The greatest impact could be a reduction of timely diagnosis and treatment, and deaths due to TB could increase by up to 20% over next 5 years [24]. Of additional concern is that the model of health service delivery could be affected in the long run. WHO has advised using digital platforms to support essential health service delivery [25], which could reduce the contact between patients and DOT providers to avoid infection during and after the COVID-19 pandemic.

In addition, while not the primary focus of this study, the increased percentage in elderly and bacteriologically confirmed TB was found to be significantly related to unfavourable treatment outcomes. Similar results for elderly people with TB were found in other studies [26, 27], suggesting that elderly people need more interventions to improve the treatment outcomes. A study from India found that patients with initial sputum of 3 + grade was significantly associated with poor treatment outcome compared with those having sputum of scanty to 2 + grade [28], but the association between treatment outcome and bacteriologically confirmed TB needs to be confirmed by further studies.

### Implications for policy and practice

In future studies, it would be worthwhile to further examine the consistency of the observation in this study with individual longitudinal data. More perspective studies are needed to confirm the effect of using EMM on TB treatment outcomes.

Furthermore, with the development of the technology, EMMs that provide real-time data could be an option, especially in some economically developed regions. We suggest doing operational research under programmatic conditions in the future.

### Limitations

Despite the strengths of the present study, there are some limitations. A potential limitation was that we relied on secondary programme data, and it is possible there were errors in recordings. The limitations of the ecological design should also be taken into account, as we can’t determine a potential direct cause and effect relationship between the increasing coverage of EMM and the improved TB treatment outcomes. Another potential limitation of this study was the absence of a controlled district, as all the 138 counties were implementing EMM, and we couldn’t evaluate the impact of other interventions on the treatment outcomes. Finally, we didn’t conduct the cost-effectiveness analysis to compare the cost of EMMS with other methods.

## Conclusions

Under programmatic settings, we found a statistically significant effect between increasing coverage of EMM and treatment success proportion at the county level. More prospective studies are needed to confirm the effect of using EMM on TB treatment outcomes. We suggest doing operational research on EMMs that provides real-time data under programmatic conditions in the future.

## Supplementary Information

**Additional file 1.** Treatment regimens for people with TB used in China (2018–19).

**Additional file 2.** Picture of the EMM device used in the 138 counties of China.

**Additional file 3.** Details of the 138 counties that implemented EMMs in China.

**Additional file 4.** The stepwise scaling up of EMMs among the 138 counties in China.

**Additional file 5.** Operational definition of TB treatment outcomes used in China (2018–19).

**Additional file 6.** Dataset and codebook.

## Data Availability

The dataset along with the codebook are provided in Additional file 6.

## Abbreviations

DOT: Directly observed therapy
EMM: Electronic medication monitor
EMMIMS: EMM information management system
SAT: Self-administered therapy
SMS: Short message service
TB: Tuberculosis
TBIMS: TB information management system
*CI*: Confidence interval
*RR*: Relative risk
