# Supplementary material for: Effect of using electronic medication monitors on tuberculosis treatment outcomes in China: a longitudinal ecological study
Source: Infect Dis Poverty. 2021 Mar 17;10:29. doi: 10.1186/s40249-021-00818-3 (PMC7967105; doi:10.1186/s40249-021-00818-3)
Supplement: Supplementary file 1 — Additional file 1. Treatment regimens for people with TB used in China (2018–19). [file 40249_2021_818_MOESM1_ESM.docx]

**Additional file 1: Treatment regimens for people with TB used in China (2018–19)**

| **Type** | **Regimen** |
| --- | --- |
| New TB | 2HRZE/4HR |
| Previously treated TB | 2HRZES/6HRE or 3HRZE/6HRE |

Abbreviations: E, ethambutol; H, isoniazid; R, rifampicin; S, streptomycin; TB, tuberculosis; Z, pyrazinamide.
