# Supplementary material for: Effect of using electronic medication monitors on tuberculosis treatment outcomes in China: a longitudinal ecological study
Source: Infect Dis Poverty. 2021 Mar 17;10:29. doi: 10.1186/s40249-021-00818-3 (PMC7967105; doi:10.1186/s40249-021-00818-3)
Supplement: Supplementary file 2 — Additional file 2. Picture of the EMM device used in the 138 counties of China. [file 40249_2021_818_MOESM2_ESM.docx]

**Additional file 2: Picture of the EMM device used in the 138 counties of China
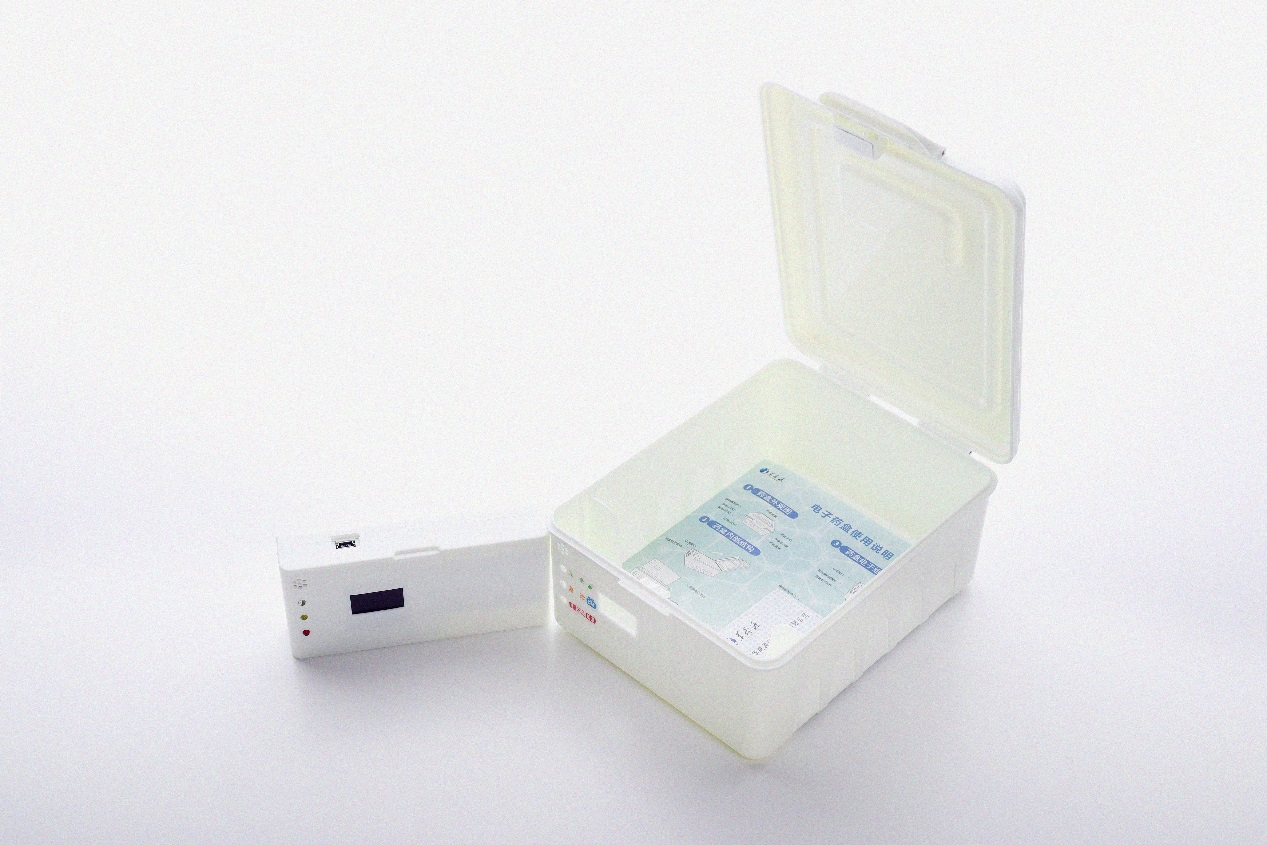
**

**Abbreviation: EMM, electronic medication monitor.**
