# Supplementary material for: Effect of using electronic medication monitors on tuberculosis treatment outcomes in China: a longitudinal ecological study
Source: Infect Dis Poverty. 2021 Mar 17;10:29. doi: 10.1186/s40249-021-00818-3 (PMC7967105; doi:10.1186/s40249-021-00818-3)
Supplement: Supplementary file 3 — Additional file 3. Details of the 138 counties that implemented EMMs in China. [file 40249_2021_818_MOESM3_ESM.docx]

**Additional file 3: Details of the 138 counties that implemented EMMs in China**

| **Region** | **Province** | **Number of counties** | **Name of the prefecture** | **Name of counties** |
| --- | --- | --- | --- | --- |
| West | Ningxia | 22 | Guyuan | Jingyuan, Longde, Pengyang, Yuanzhou, Xiji |
|  |  |  | Shizuishan | Huinong, Pingluo, Dawukou |
|  |  |  | Wuzhong | Yanchi, Hongsipu, Qingtongxia,Tongxin,Litong |
|  |  |  | Yinchuan | Lingwu,Yongning,Helan,Xixia,Xingqing,Jinfeng |
|  |  |  | Zhongwei | Zhongning, Shapotou, Haiyuan |
| Eastern | Zhejiang | 65 | Huzhou | Deqing, Anji, Changxing, Wuxing, Nanxun |
|  |  |  | Jiaxing | Haiyan, Pinghu, Tongxiang, Jiashan, Haining, Nanhu, Xiuzhou |
|  |  |  | Jinhua | Panan, Wuyi, Lanxi, Pujiang, Wucheng, Jindong, Dongyang, Yongkang, Yiwu |
|  |  |  | Lishui | Yunhe, Jingning, Qingyuan, Suichang, Longyuan, Jinyun, Songyang, Qingtian, Liandu |
|  |  |  | Ningbo | Zhenhai, Xiangshan, Fenghua, Yinzhou, Beilun, Cixi, Yuyao, Ninghai, Haishu, Jiangbei |
|  |  |  | Guzhou | Qujiang, Changshan, Longyou, Kaihua, Jiangshan, Kecheng |
|  |  |  | Shaoxing | Shangyu, Keqiao, Xinchang, Shengzhou, Zhuji, Yuecheng |
|  |  |  | Taizhou | Xianju, Sanmen, Tiantai, Luqiao, Jiangjiang, Huangyan, Linhai, Wenling, Yuhuan |
|  |  |  | Zhoushan | Shengsi, Dinghai, Putuo, Daishan |
| Middle | Jilin | 51 | Baicheng | Taobei, Taonan, Daan, Tongyu, Zhenlai |
|  |  |  | Baishan | Changbai, Jingyu, Jiangyuan, Hunjiang, Fusong, Linjiang |
|  |  |  | Liaoyuan | Xian, Longshan, Dongfeng, Dongliao |
|  |  |  | Siping | Lishu, Tiedong, Tiexi, Shuangliao, Yitong, Gongzhuling |
|  |  |  | Songyuan | Fuyu, Changling, Ningjiang, Qianguoerluosi, Qianan |
|  |  |  | Tonghua | Dongchang, Erdaojiang, Liuhe, Huinan, Tonghua, Jian, Meihekou |
|  |  |  | Yanbian | Longjiang, Helong, Tumen, Yanji, Wangqing, Antu, Dunhua, Huichun |
|  |  |  | Changchun | Nongan, Dehui, Yushu, Shuangyang, Nanguan, Erdao, Chaoyang, Lvyuan, Jiutai, Kuancheng, |

Abbreviation: EMM, electronic medication monitor.
