# Supplementary material for: Effect of using electronic medication monitors on tuberculosis treatment outcomes in China: a longitudinal ecological study
Source: Infect Dis Poverty. 2021 Mar 17;10:29. doi: 10.1186/s40249-021-00818-3 (PMC7967105; doi:10.1186/s40249-021-00818-3)
Supplement: Supplementary file 4 — Additional file 4. The stepwise scaling up of EMMs among the 138 counties in China. [file 40249_2021_818_MOESM4_ESM.docx]

**Additional file 4: The stepwise scaling up of EMMs among the 138 counties in China**

*(Grey areas represent the quarters when the county was using EMM)*

| **County name** | **2017.Q2** | **2017.Q3** | **2017.Q4** | **2018.Q1** | **2018.Q2** | **2018.Q3** | **2018.Q4** | **2019.Q1** | **2019.Q2** |
| --- | --- | --- | --- | --- | --- | --- | --- | --- | --- |
| **Quzhou-changshan** |  |  |  |  |  |  |  |  |  |
| **Quzhou-longyou** |  |  |  |  |  |  |  |  |  |
| **Quzhou-qujiang** |  |  |  |  |  |  |  |  |  |
| **Shuizuishan-dawukou** |  |  |  |  |  |  |  |  |  |
| **Tonghua-dongchang** |  |  |  |  |  |  |  |  |  |
| **Tonghua-erdaojiang** |  |  |  |  |  |  |  |  |  |
| **Tonghua-huinan** |  |  |  |  |  |  |  |  |  |
| **Meihekou** |  |  |  |  |  |  |  |  |  |
| **Tonghua-tonghua** |  |  |  |  |  |  |  |  |  |
| **Yinchuan-lingwu** |  |  |  |  |  |  |  |  |  |
| **Baicheng-daan** |  |  |  |  |  |  |  |  |  |
| **Baishan-fusong** |  |  |  |  |  |  |  |  |  |
| **Baishan-linjiang** |  |  |  |  |  |  |  |  |  |
| **Jiaxing-haiyan** |  |  |  |  |  |  |  |  |  |
| **Jiaxing-pinghu** |  |  |  |  |  |  |  |  |  |
| **Jiaxing-tongxiang** |  |  |  |  |  |  |  |  |  |
| **Liaoyuan-xian** |  |  |  |  |  |  |  |  |  |
| **Liaoyuan-longshan** |  |  |  |  |  |  |  |  |  |
| **Quzhou-jiangshan** |  |  |  |  |  |  |  |  |  |
| **Quzhou-kaihua** |  |  |  |  |  |  |  |  |  |
| **Quzhou-kecheng** |  |  |  |  |  |  |  |  |  |
| **Shizuishan-pingluo** |  |  |  |  |  |  |  |  |  |
| **Siping-tiedong** |  |  |  |  |  |  |  |  |  |
| **Siping-tiexi** |  |  |  |  |  |  |  |  |  |
| **Tonghua-jian** |  |  |  |  |  |  |  |  |  |
| **Tonghua-liuhe** |  |  |  |  |  |  |  |  |  |
| **Wuzhong-yanchi** |  |  |  |  |  |  |  |  |  |
| **Yanbian-dunhua** |  |  |  |  |  |  |  |  |  |
| **Yanbian-huichun** |  |  |  |  |  |  |  |  |  |
| **Yanbian-yanji** |  |  |  |  |  |  |  |  |  |
| **Yinchuan-yongning** |  |  |  |  |  |  |  |  |  |
| **Changchun-erdao** |  |  |  |  |  |  |  |  |  |
| **Changchun-lvyuan** |  |  |  |  |  |  |  |  |  |
| **Baicheng-tongyu** |  |  |  |  |  |  |  |  |  |
| **Baishan-hunjiang** |  |  |  |  |  |  |  |  |  |
| **Huzhou-deqing** |  |  |  |  |  |  |  |  |  |
| **Jiaxing-jiashan** |  |  |  |  |  |  |  |  |  |
| **Jinhua-pujiang** |  |  |  |  |  |  |  |  |  |
| **Jinhua-wuyi** |  |  |  |  |  |  |  |  |  |
| **Jinhua-wucheng** |  |  |  |  |  |  |  |  |  |
| **Jinhua-jindong** |  |  |  |  |  |  |  |  |  |
| **Jinhua-yiwu** |  |  |  |  |  |  |  |  |  |
| **Lishui-jingning** |  |  |  |  |  |  |  |  |  |
| **Lishui-longquan** |  |  |  |  |  |  |  |  |  |
| **Lishui-qingtian** |  |  |  |  |  |  |  |  |  |
| **Lishui-songyang** |  |  |  |  |  |  |  |  |  |
| **Lishui-suichang** |  |  |  |  |  |  |  |  |  |
| **Lishui-yunhe** |  |  |  |  |  |  |  |  |  |
| **Shaoxing-keqiao** |  |  |  |  |  |  |  |  |  |
| **Shaoxing-zhuji** |  |  |  |  |  |  |  |  |  |
| **Gongzhuling** |  |  |  |  |  |  |  |  |  |
| **Siping-yitong** |  |  |  |  |  |  |  |  |  |
| **Songyuan-fuyu** |  |  |  |  |  |  |  |  |  |
| **Songyuan-qianluoerluosi** |  |  |  |  |  |  |  |  |  |
| **Songyuan-changling** |  |  |  |  |  |  |  |  |  |
| **Taizhou-huangyan** |  |  |  |  |  |  |  |  |  |
| **Taizhou-linhai** |  |  |  |  |  |  |  |  |  |
| **Taizhou-luqiao** |  |  |  |  |  |  |  |  |  |
| **Taizhou-sanmen** |  |  |  |  |  |  |  |  |  |
| **Taizhou-tiantai** |  |  |  |  |  |  |  |  |  |
| **Taizhou-yuhuan** |  |  |  |  |  |  |  |  |  |
| **Wuzhong-tongxin** |  |  |  |  |  |  |  |  |  |
| **Yanbian-longjing** |  |  |  |  |  |  |  |  |  |
| **Changchun-kuancheng** |  |  |  |  |  |  |  |  |  |
| **Changchun-nanguan** |  |  |  |  |  |  |  |  |  |
| **Huzhou-anji** |  |  |  |  |  |  |  |  |  |
| **Huzhou-wuxing** |  |  |  |  |  |  |  |  |  |
| **Huzhou-nanxun** |  |  |  |  |  |  |  |  |  |
| **Huzhou-changxing** |  |  |  |  |  |  |  |  |  |
| **Jiaxing-haining** |  |  |  |  |  |  |  |  |  |
| **Jinhua-dongyang** |  |  |  |  |  |  |  |  |  |
| **Jinhua-lanxi** |  |  |  |  |  |  |  |  |  |
| **Lishui-liandu** |  |  |  |  |  |  |  |  |  |
| **Lishui-qingyuan** |  |  |  |  |  |  |  |  |  |
| **Shaoxing-shengzhou** |  |  |  |  |  |  |  |  |  |
| **Shizuishan-huinong** |  |  |  |  |  |  |  |  |  |
| **Taizhou-wenling** |  |  |  |  |  |  |  |  |  |
| **Yinchuan-xixia** |  |  |  |  |  |  |  |  |  |
| **Yichuan-xingqing** |  |  |  |  |  |  |  |  |  |
| **Yinchuan-jinfeng** |  |  |  |  |  |  |  |  |  |
| **Jinhua-panan** |  |  |  |  |  |  |  |  |  |
| **Jinhua-yongkang** |  |  |  |  |  |  |  |  |  |
| **Liaoyuan-dongliao** |  |  |  |  |  |  |  |  |  |
| **Taizhou-jiaojiang** |  |  |  |  |  |  |  |  |  |
| **Taizhou-xianju** |  |  |  |  |  |  |  |  |  |
| **Wuzhong-litong** |  |  |  |  |  |  |  |  |  |
| **Wuzhong-qingtongxia** |  |  |  |  |  |  |  |  |  |
| **Yinchuan-helan** |  |  |  |  |  |  |  |  |  |
| **Changchun-chaoyang** |  |  |  |  |  |  |  |  |  |
| **Baishan-jiangyuan** |  |  |  |  |  |  |  |  |  |
| **Jiaxing-nanhu** |  |  |  |  |  |  |  |  |  |
| **Jiaxing-xiuzhou** |  |  |  |  |  |  |  |  |  |
| **Lishui-jinyun** |  |  |  |  |  |  |  |  |  |
| **Shaoxing-xinchang** |  |  |  |  |  |  |  |  |  |
| **Shaoxing-yuecheng** |  |  |  |  |  |  |  |  |  |
| **Wuzhong-hongsipu** |  |  |  |  |  |  |  |  |  |
| **Changchun-nongan** |  |  |  |  |  |  |  |  |  |
| **Changchun-shuangyang** |  |  |  |  |  |  |  |  |  |
| **Changchun-yushu** |  |  |  |  |  |  |  |  |  |
| **Zhoushan-shengsi** |  |  |  |  |  |  |  |  |  |
| **Baishan-jingyu** |  |  |  |  |  |  |  |  |  |
| **Ningbo-beilun** |  |  |  |  |  |  |  |  |  |
| **Ningbo-cixi** |  |  |  |  |  |  |  |  |  |
| **Ningbo-fenghua** |  |  |  |  |  |  |  |  |  |
| **Ningbo-ninghai** |  |  |  |  |  |  |  |  |  |
| **Ningbo-xiangshan** |  |  |  |  |  |  |  |  |  |
| **Ningbo-yinzhou** |  |  |  |  |  |  |  |  |  |
| **Ningbo-zhenhai** |  |  |  |  |  |  |  |  |  |
| **Shaoxing-shangyu** |  |  |  |  |  |  |  |  |  |
| **Songyuan-qianan** |  |  |  |  |  |  |  |  |  |
| **Changchun-jiutai** |  |  |  |  |  |  |  |  |  |
| **Zhongwei-shapotou** |  |  |  |  |  |  |  |  |  |
| **Zhoushan-dinghai** |  |  |  |  |  |  |  |  |  |
| **Zhoushan-putuo** |  |  |  |  |  |  |  |  |  |
| **Baicheng-taobei** |  |  |  |  |  |  |  |  |  |
| **Baicheng-taonan** |  |  |  |  |  |  |  |  |  |
| **Baicheng-zhenlai** |  |  |  |  |  |  |  |  |  |
| **Baishan-changbai** |  |  |  |  |  |  |  |  |  |
| **Liaoyuan-dongfeng** |  |  |  |  |  |  |  |  |  |
| **Ningbo-yuyao** |  |  |  |  |  |  |  |  |  |
| **Siping-lishu** |  |  |  |  |  |  |  |  |  |
| **Siping-shuangliao** |  |  |  |  |  |  |  |  |  |
| **Songyuan-ningjiang** |  |  |  |  |  |  |  |  |  |
| **Yanbian-antu** |  |  |  |  |  |  |  |  |  |
| **Yanbian-helong** |  |  |  |  |  |  |  |  |  |
| **Yanbian-tumen** |  |  |  |  |  |  |  |  |  |
| **Yanbian-wangqing** |  |  |  |  |  |  |  |  |  |
| **Changchun-dehui** |  |  |  |  |  |  |  |  |  |
| **Zhongwei-zhongning** |  |  |  |  |  |  |  |  |  |
| **Guyuan-longde** |  |  |  |  |  |  |  |  |  |
| **Guyuan-pengyang** |  |  |  |  |  |  |  |  |  |
| **Ningbo-haishu** |  |  |  |  |  |  |  |  |  |
| **Ningbo-jingbei** |  |  |  |  |  |  |  |  |  |
| **Zhoushan-daishan** |  |  |  |  |  |  |  |  |  |
| **Guyuan-jingyuan** |  |  |  |  |  |  |  |  |  |
| **Guyuan-xiji** |  |  |  |  |  |  |  |  |  |
| **Guyuan-yuanzhou** |  |  |  |  |  |  |  |  |  |
| **Zhongwei-haiyuan** |  |  |  |  |  |  |  |  |  |
