# Supplementary material for: Effect of using electronic medication monitors on tuberculosis treatment outcomes in China: a longitudinal ecological study
Source: Infect Dis Poverty. 2021 Mar 17;10:29. doi: 10.1186/s40249-021-00818-3 (PMC7967105; doi:10.1186/s40249-021-00818-3)
Supplement: Supplementary file 5 — Additional file 5. Operational definition of TB treatment outcomes used in China (2018–19). [file 40249_2021_818_MOESM5_ESM.docx]

**Additional file 5: Operational definition of TB treatment outcomes used in China (2018–19)**

| **Outcome** | **Definition*** |
| --- | --- |
| Cured | A pulmonary TB patient with bacteriologically confirmed TB at the beginning of treatment who was smear- or culture-negative in the last month of treatment and on at least one previous occasion. |
| Treatment completed | A TB patient who completed treatment without evidence of failure BUT with no record to show that sputum smear or culture results in the last month of treatment and on at least one previous occasion were negative, either because tests were not done or because results are unavailable. |
| Treatment failed | A TB patient whose sputum smear or culture is positive at month 5 or later during treatment. |
| Died | A TB patient who dies for any reason before starting or during the course of treatment. |
| Lost to follow-up | A TB patient who did not start treatment or whose treatment was interrupted for 2 consecutive months or more. |
| Not evaluated | A TB patient for whom no treatment outcome is assigned. This includes patients “transferred out” to another treatment unit as well as patients for whom the treatment outcome is unknown to the reporting unit. |
| Treatment success | The sum of cured and treatment completed. |
| Unfavourable outcome* | All outcomes other than cured and treatment completed. |

Source: World Health Organizations. Definitions and reporting framework for tuberculosis: 2013 revision (updated December 2014).

*If patient is transferred to MDR-TB care during TB treatment and there is evidence of patient registering in the prefecture-level MDR-TB centre, then patient will be excluded from this cohort. If there is no evidence of patient registering in the prefecture level MDR-TB centre, then patient will be included in this cohort and reported under unfavourable outcomes. Any patient who is still on treatment will included under favourable outcomes.
